# Supplementary material for: Efficacy and safety of Xuebijing injection for radiation pneumonitis: A meta-analysis
Source: PLoS One. 2022 Jun 1;17(6):e0268819. doi: 10.1371/journal.pone.0268819 (PMC9159617; doi:10.1371/journal.pone.0268819)
Supplement: S1 File — The Efficacy and Safety of Xuebijing Injection in the Treatment of Radiation Pneumonitis: A Protocol for Systematic Review and Meta-Analysis. (PDF) [file pone.0268819.s002.pdf]

International Platform of Registered Systematic Review and Meta-analysis Protocols

INPLASY PROTOCOL

To cite: Li et al. The Efficacy and Safety of Xuebijing Injection in the Treatment of Radiation Pneumonitis : A Protocol for Systematic Review and Meta-Analysis. Inplasy protocol 2020120037. doi: 10.37766/inplasy2020.12.0037

Received: 06 December 2020

Published: 07 December 2020

Corresponding author:
Wei Hou

houwei1964@163.com

Author Affiliation:
Guang'an men Hospital, China Academy of Chinese Medical Sciences

Support: **Natural Science Foundation(CN).**

Review Stage at time of this submission: **The review has not yet started.**

Conflicts of interest:
We have no conflict of interest.

The Efficacy and Safety of Xuebijing Injection in the Treatment of Radiation Pneumonitis: A Protocol for Systematic Review and Meta-Analysis

Li, Z[1]; Wang, D[2]; Wang, S[3]; Wang, X[4]; Li, Y[5]; Gui, Y[6]; Dong, J[7]; Zhang, Y[8]; Hou, W[9].

Review question / Objective: P: patients with Radiation Pneumonitis; I: Xuebijing injection along with glucocorticoids, bronchodilators, antibiotics, oxygen therapy and respiratory support treatment; C: glucocorticoids, bronchodilators, antibiotics, oxygen therapy and respiratory support treatment; O: total effective rate, C-reactive protein, tumor necrosis factor alpha, interleukin-6, interleukin-10, adverse events; S: randomized controlled trials.
Condition being studied: At present, the treatment of Radiation Pneumonitis (RP) is still a clinical problem. Although a variety of drugs such as glucocorticoids and antibiotics are used for RP treatment, side effects remain to be inevitable. Xuebijing injection(XBJ), a Chinese herbal injection , has been widely used in Radiation Pneumonitis treatment, but there is no published systematic review to evaluate its efficacy and safety.

INPLASY registration number: This protocol was registered with the International Platform of Registered Systematic Review and Meta-Analysis Protocols (INPLASY) on 07 December 2020 and was last updated on 07 December 2020 (registration number INPLASY2020120037).

INTRODUCTION

Review question / Objective: P: patients with Radiation Pneumonitis; I: Xuebijing injection along with glucocorticoids, bronchodilators, antibiotics, oxygen therapy and respiratory support treatment; C: glucocorticoids, bronchodilators, antibiotics, oxygen therapy and respiratory support treatment; O: total effective rate, C-reactive protein, tumor necrosis factor

Li et al. Inplasy protocol 2020120037. doi:10.37766/inplasy2020.12.0037 Downloaded from https://inplasy.com/inplasy-2020-12-0037/

alpha, interleukin-6, interleukin-10, adverse events; S: randomized controlled trials.

Condition being studied: At present, the treatment of Radiation Pneumonitis (RP) is still a clinical problem. Although a variety of drugs such as glucocorticoids and antibiotics are used for RP treatment, side effects remain to be inevitable. Xuebijing injection(XBJ), a Chinese herbal injection , has been widely used in Radiation Pneumonitis treatment, but there is no published systematic review to evaluate its efficacy and safety.

METHODS

Search strategy: #1 Radiation Pneumonitis[MESH]; #2 (Radiation Pneumonitides) OR (Pneumonitides, Radiation) OR (Pneumonia, Radiation) OR (Pneumonias, Radiation) OR (Radiation Pneumonias) OR (Pneumonitis, Radiation) OR (Radiation Pneumonia) OR (Fibrosis, Radiation) OR (Radiation Fibrosis) OR (Radiation induced lung injury) OR (Radiation induced pneumonitis); #3 (XUEBIJING) OR (XUE-BI-JING) OR (XUEBIJING injection) OR (XUE-BI-JING injection); #4 (Randomized Controlled Trials[Mesh])OR(Clinical Trials, Randomized) OR (Trials, Randomized Clinical) OR (Controlled Clinical Trials, Randomized) OR (RCT); #5 #1 AND #2 AND #3 AND #4.

Participant or population: Patients with Radiation Pneumonitis.

Intervention: Xuebijing injection along with glucocorticoids, bronchodilators, antibiotics, oxygen therapy and respiratory support treatment.

Comparator: Glucocorticoids, bronchodilators, antibiotics, oxygen therapy and respiratory support treatment lucocorticoids, bronchodilators, antibiotics, oxygen therapy and respiratory support treatment if necessary.

Study designs to be included: Only RCTs will be eligible for inclusion regardless of the languages. It will not include animal experiments, case reports, non-clinical researches, commentaries, repeated publications, etc. RCTs with incomplete and unavailable important data will not be included.

Eligibility criteria: 1. Types of studies. Only RCTs will be eligible for inclusion regardless of the languages. It will not include animal experiments, case reports, non-clinical researches, commentaries, repeated publications, etc. RCTs with incomplete and unavailable important data will be excluded. 2. Type of participants. The study will recruit participants over 18 years of age who met the histological or pathological or clinical diagnostic criteria for Radiation Pneumonitis. There will be no restrictions on gender, nationality, race, education and job. 3. Type of interventions. The interventions will contain two groups: experimental group and control group.The interventions of control group will include glucocorticoids, bronchodilators, antibiotics, oxygen therapy and respiratory support treatment if necessary. The interventions of experimental group will include the interventions of control group and Xuebijing injection. 4. Primary outcomes. The primary outcome is the total effective rate. In brief, the total effective rate equals the cure rate and the effective rate. 5. Secondary outcomes. The secondary outcome will consist of inflammation-related indicators such as C-reactive protein , tumor necrosis factor alpha, interleukin-6, interleukin-10, etc. and adverse events.

Information sources: We will comprehensively search 7 databases: PubMed, Cochrane Library, EMBASE, China National Knowledge Infrastructure (CNKI), WANFANG database, SinoMED and China Science and Technology Journal Database (VIP). There is no limitation of the language.The time interval for literature searching will be from inception of the library to October 1, 2020.

Main outcome(s): Total effective rate, C-reactive protein, tumor necrosis factor alpha, interleukin-6, interleukin-10.

Li et al. Inplasy protocol 2020120037. doi:10.37766/inplasy2020.12.0037 Downloaded from https://inplasy.com/inplasy-2020-12-0037/

Quality assessment / Risk of bias analysis: Two reviewers will use the “risk of bias assessment tool” of the Cochrane Handbook to evaluate the risk of bias of the included RCTs. The evaluation criteria include the method of random sequence generation, allocation concealment, blinding of patients, personnel and assessors, incomplete outcome data, selective reporting, and other sources of bias. Any disagreement will be resolved by the third reviewer.

Strategy of data synthesis: Data synthesis and analysis will be conducted by Revman 5.3 software. Different data types will be processed in different ways: continuous data and dichotomous data will be evaluated by standard mean difference (SMD) with 95% confidence interval (95% CI) and rate ratio (RR) with 95% CI respectively. The heterogeneity will judged by the I^2 value. For $I^2$50%, the fixed effects model and the random effects model will be used respectively.

Subgroup analysis: We will conduct subgroup analysis based on interventions, participants characteristics, and outcome measurement if the included articles are at least 10.

Sensibility analysis: In the sensitivity analysis, we will remove each study in turn to observe the impact on the overall results.

Country(ies) involved: China.

Keywords: Radiation Pneumonitis, Xuebijing injection, Efficacy and safety, Randomized controlled trials, Meta-analysis.

Contributions of each author:
Author 1 - Zheng Li wrote the protocol and revised the manuscript.
Email: lizheng_gam@163.com
Author 2 - Dandan Wang wrote the protocol and revised the manuscript.
Email: wdd19940416@163.com
Author 3 - Shuo Wang developed the criteria and conducted statistical methods.
Email: 18701522023@163.com
Author 4 - Wueqian Wang designed and conceptualized the study.
Email: wxq791875354@126.com
Author 5 - Yuxiao Li developed the criteria and conducted statistical methods.
Email: liyuxiao2593@126.com
Author 6 - Yuerong Gui advised on protocol design.
Email: 1172628045@qq.com
Author 7 - Jun Dong advised on protocol design.
Email: 2577930287@qq.com
Author 8 - Ying Zhang designed and conceptualized the study.
Email: zylzy501@163.com
Author 9 - Wei Hou designed and conceptualized the study.
Email: houwei1964@163.com

Li et al. Inplasy protocol 2020120037. doi:10.37766/inplasy2020.12.0037 Downloaded from https://inplasy.com/inplasy-2020-12-0037/
