# Supplementary material for: Efficacy and safety of Xuebijing injection for radiation pneumonitis: A meta-analysis
Source: PLoS One. 2022 Jun 1;17(6):e0268819. doi: 10.1371/journal.pone.0268819 (PMC9159617; doi:10.1371/journal.pone.0268819)
Supplement: Attachment — Submitted filename: PLOS one review _JSG.docx [file pone.0268819.s003.docx]

The manuscript submitted by the authors describe about the metaanalysis of 8 studies describing an agent Xuibijing as a radiation protector against radiation pneumonitis.

The authors have only taken randomised clinical trial for the purpose of metananalysis and have omitted studies which were preclinical in nature and studies which were retrospective.

The authors have followed the PRISMA guidelines for the metaanalysis and have properly done the risk bias assessment of all the studies as represented by the traffic light plot.

The authors have used appropriate statististical tests for analysing the data from the RCT’s.

Although the forest plots show a favourable response with the use of the experimental drug in terms of various diagnostic metrics, even then the authors do not recommend the use of the agent in question for radiation pneumonits.

The manuscript is well written and adheres strictly to the guidelines for reporting meta-analysis. Even though the metaanalysis can be termed as negative against the use of the experimental agent, I would still recommend the publication of the study after major modifications.

**Comments**

1. The authors in the discussion section need to elaborate a bit more on this agent as its mechanism of action, its pharmacology, kinetics and its toxicities have not been extensively reported given that it is a Chinese medicine and the readers outside China will not have much knowledge about it.
2. What is a bit surprising to me is that the clinical end points and majority of the biological markers (inflammatory cytokines) showed an improvement on the use of combination of the experimental drug + conventional therapy as opposed to the conventional therapy alone except for IL10 which is a proinflammatory cytokine which showed an increased levels which is a bit counterintuitive? An explanation needs to b e give regarding this phenomenon in the discussion section.
3. In the methodology section: the authors should write in detail if any of the studies did an objective assessment for radiation pneumonitis.
4. The authors have shown in their forest plots the results of the RCT’s favouring the use of xuibijing against RP as none of the plots were straddling the unity line. However, based on the limitation s of the RCT’s, the authors in their concluding remarks have not recommended the use of this agent against RP. I would expect the authors to clearly annotate the forest plots and expand the legends of figure 3. I would also want the authors to clearly mention the diagnostic metrics they used in the forest plots.(was it the Hazard ratio or the odds ratio?)
5. The authors also mention that none of the studies mentioned anything about AE’s & SAE’s in the limitation of the studies. In this last paragraph, the authors mention about TCM injection. The authors write in detail what TCM stands for as it has not been mentioned previously in the manuscript.
6. The authors mentioned that they did not do a funnel plot as the number of studies were less than 10. However, it will be worthwhile to do the HSROC curves to depict the extent of heterogeinity of each study.
